# Modulation of Pain Sensitivity by Chronic Consumption of Highly Palatable Food Followed by Abstinence: Emerging Role of Fatty Acid Amide Hydrolase

**DOI:** 10.3389/fphar.2020.00266

**Published:** 2020-03-16

**Authors:** Carlo Cifani, Carmen Avagliano, Emanuela Micioni Di Bonaventura, Maria Elena Giusepponi, Carmen De Caro, Claudia Cristiano, Giovanna La Rana, Luca Botticelli, Adele Romano, Antonio Calignano, Silvana Gaetani, Maria Vittoria Micioni Di Bonaventura, Roberto Russo

**Affiliations:** ^1^ School of Pharmacy, Pharmacology Unit, University of Camerino, Camerino, Italy; ^2^ Department of Pharmacy, “Federico II” University of Naples, Naples, Italy; ^3^ Department of Physiology and Pharmacology “V. Erspamer,” Sapienza University of Rome, Rome, Italy

**Keywords:** obesity, animal models, brain, high-fat diet, pain

## Abstract

There is a strong relationship between palatable diet and pain sensitivity, and the cannabinoid and opioid systems might play an important role in this correlation. The palatable diet used in many animal models of obesity is the cafeteria (CAF) diet, based on human food with high sugar, salt, and fat content. In this study, we investigated whether long-term exposure to a CAF diet could modify pain sensitivity and explored the role of the cannabinergic system in this modification. Male Sprague–Dawley rats were divided into two groups: one fed with standard chow only (CO) and the other with extended access (EA) to a CAF diet. Hot plate and tail flick tests were used to evaluate pain sensitivity. At the end of a 40-day CAF exposure, EA rats showed a significant increase in the pain threshold compared to CO rats, finding probably due to up-regulation of CB1 and mu-opioid receptors. Instead, during abstinence from palatable foods, EA animals showed a significant increase in pain sensibility, which was ameliorated by repeated treatment with a fatty acid amide hydrolase inhibitor, PF-3845 (10 mg/kg, intraperitoneally), every other day for 28 days. *Ex vivo* analysis of the brains of these rats clearly showed that this effect was mediated by mu-opioid receptors, which were up-regulated following repeated treatment of PF-3845. Our data add to the knowledge about changes in pain perception in obese subjects, revealing a key role of CB1 and mu-opioid receptors and their possible pharmacological crosstalk and reinforcing the need to consider this modulation in planning effective pain management for obese patients.

## Introduction

The modern diet, or so-called Western diet, contains many sweetened beverages and foods rich in sugar, salt, and fat (Deshmukh-Taskar et al., 2009). The chronic consumption of energy-rich foods is motivated not only by their nutritional value but also the hedonic pleasure they impart (de Macedo et al., 2016), and thus subjects break energy homeostasis (Ghanemi et al., 2018), overeat, and become obese (Shafat et al., 2009).

To explain the lack of control of food intake, many studies hypothesized the concept of food addiction (Avena and Gold, 2011; Micioni Di Bonaventura et al., 2012; D'Addario et al., 2014), as these subjects seem to have an uncontrollable urge to eat large amounts of palatable foods based on a state of reward hyposensitivity comparable to that of drug addiction (Johnson and Kenny, 2010; Avena and Gold, 2011). In fact, in a dynamic similar to that of substance abuse, the eaters develop a compulsive-feeding behavior in search of reward, despite the negative consequences (Gold et al., 2003). Several studies demonstrated that rodents have such high motivation to obtain palatable food that they even expose themselves to extreme cold, noxious heat pain, or aversive foot shock (Cabanac and Johnson, 1983; Foo and Mason, 2005; Oswald et al., 2011). Moreover, in another study, mice fed with palatable food were much more likely to spend time in an aversive environment in order to obtain pleasant food compared to mice without experience of this diet (Teegarden and Bale, 2007). Intake of sucrose solutions and dietary fat alters pain sensitivity in animals, another finding that points to a relationship between endogenous opioid peptides and palatable foods (Davis et al., 1956; Blass et al., 1987; Klein and Green, 1988; Markskaufman et al., 1988; Shide and Blass, 1989; Kanarek et al., 1991; Frye et al., 1992). Human studies have produced similar observations. Consumption of a sucrose solution alleviated pain reactions and decreased crying after painful procedures in newborns (Blass and Hoffmeyer, 1991; Smith et al., 1992; Blass and Shide, 1994; Smith and Blass, 1996; Herschel et al., 1998; Overgaard and Knudsen, 1999). In children (Miller et al., 1994) and adult male subjects (Kakeda et al., 2008), sweet substances increased pain threshold, whereas a number of other works have observed that in adults the intake of palatable foods may have analgesic properties (Miller et al., 1994; Mercer and Holder, 1997; Lewkowski et al., 2003). Taken together, these results suggest that diet can modulate the action of central nervous system (CNS) mechanisms involved in pain sensitivity. This correlation extended to obesity and pain could have important implications for the treatment of pain in obese human subjects.

Different works have examined pain perception in obese animals and humans compared to nonobese subjects, but the debate is still open (Chin et al., 2019). A recent systematic review (Torensma et al., 2016) included two studies suggesting that obese subjects have a lower pain threshold than nonobese subjects (Pradalier et al., 1981; McKendall and Haier, 1983), but four studies indicating that obese subjects have a higher one (Zahorska-Markiewicz et al., 1988; Miscio et al., 2005; Maffiuletti et al., 2011; Dodet et al., 2013). Other findings concluded that body mass index and body fat may influence the pain threshold (Bohnert et al., 2013; Price et al., 2013; Tashani et al., 2017; Torensma et al., 2017), and the intensity of the perception of stimuli changes proportionally with the levels of subcutaneous fat in the affected body area. Price et al. (2013) reported that obese people have a higher pain threshold in the abdominal area compared to the hands and forearms. Tashani et al. (2017) showed different results: obese individuals were more sensitive to pressure pain but not to thermal pain, suggesting a more complex relationship between fat distribution and pain sensitivity. In the same year, in a study comparing obese patients and control patients in their pain perception and ability to grade pain, Torensma et al. (2016) reported that in experiments obese patients revealed hypoalgesia to noxious electrical stimuli and had difficulty grading noxious thermal and electrical stimuli set between the pain threshold and the tolerance level. In summary, all these studies indicate that pain perception may be altered in obese subjects, but the variability in the findings, related to different methodologies, pain tests, and selection of participants, makes it difficult to determine appropriate pain relief strategies for such patients. In this context, the study of cannabinoids and opioids may help our understanding of the mechanisms involved in overeating and pain perception.

Cannabinoids and opioids share many pharmacological properties, including analgesia and control of hunger (Fattore et al., 2005; Micioni Di Bonaventura et al., 2013). It has been reported that the opioid antagonist naloxone inhibited antinociception induced by a cannabinoid (Manzanares et al., 1998), and another study found that the antinociceptive properties of subeffective doses of both drugs were synergistic (Massi et al., 2001).

Repeated treatment with CB1 agonist increases proenkephalin gene expression and mu-opioid receptors (Corchero et al., 1997a; Corchero et al., 1997b; Corchero et al., 1999). In feeding regulation, the hyperphagic effect of endocannabinoid and the appetite suppression resulting from the blockade of CB1 have been clearly documented (see recent reviews, Koch, 2017; Tarragon and Moreno, 2019).

Similarly, opioid receptor agonists increase the intake of high-fat diets, whereas antagonists decrease it (reviewed in (Le Foll and Goldberg, 2005; Micioni Di Bonaventura et al., 2019). The interaction between these two systems may indicate that their relationship plays a role in the rewarding value of food and in the regulation of ingestive behavior (Cota et al., 2006).

Based on these considerations, we investigated in male Sprague–Dawley rats (i) how long-term exposure to a palatable diet and then abstinence from it could modify pain sensitivity and (ii) the possible role of the cannabinergic and opioidergic systems. For this purpose, we used the “cafeteria (CAF) diet,” consisting of energy-dense foods such as cheese, processed meats, chocolate, and cookies, which reflects the variety of the unhealthy human foods and CAF is used in many animal obesity models (Sclafani and Springer, 1976; Sampey et al., 2011). Pain sensitivity was evaluated at the end of 40 days of CAF exposure, and in the middle and at the end of the abstinence period from CAF (at days 54 and 68, respectively). To assess the involvement of the cannabinergic and opioidergic systems, during the abstinence period, we used PF-3845, a selective and long half-life fatty acid amide hydrolase (FAAH) inhibitor (Ahn et al., 2009; Mileni et al., 2010), in order to increase the acylethanolamide tone. With the choice of PF-3845, we were able to avoid the undesirable side effects that are observed with direct CB1 agonists.

## Materials and Methods

### Animals

Male Sprague–Dawley rats (300–350 g, 10–11 weeks old) were purchased from Charles River Laboratories (Calco, Italy) and housed in single plastic cages (40 × 60 × 30 cm) on fresh bedding with free access to food and water. The environment was controlled with a 12-h light–dark cycle and a room temperature of 22.0°C ± 2.0°C. The animals did not show any signs of stress (except nociception-related behavior) or illness throughout the experiment. All animal procedures in this study were conducted in strict accordance with the National Institutes of Health Guide for the Care and Use of Laboratory Animals (Italian Ministry of Health, protocol n.887/2015-PR; 19/01/2017) and associated guidelines from European Communities Council Directive (2010/63/EU), as well as the ethical standards of the International Association for the Study of Pain (Zimmermann, 1983).

All behavioral experiments were performed by experimenters who were blinded to the experimental groups and treatments.

### Drugs

*N*-(piperidin-1-yl)-5-(4-chlorophenyl)-1-(2,4-dichlorophenyl)-4-methyl-1Hpyrazole-3-carboxyamide hydrochloride (SR141716A, SR1) was obtained from the National Institute on Drug Abuse; naloxone hydrochloride (Nal) was purchased from Tocris (Bristol, UK), and N-3-pyridinyl-4-[[3-[[5-(trifluoromethyl)-2-pyridinyl]oxy]phenyl]methyl]-1-piperidinecarboxamide (PF-3845) was purchased from Selleck Chemicals (Aurogene, Rome, Italy). SR1 or Nal was dissolved in PEG400 and Tween 80 2:1 (Sigma-Aldrich, Milan, Italy) and kept overnight under gentle agitation with a micro stirring bar. Before injection, sterile saline was added, so that the final concentrations of PEG400 and Tween 80 were 20% and 10% vol/vol, respectively. SR1 was administered intraperitoneally (IP) at the dose of 1 mg/kg (La Rana et al., 2006); Nal was administered IP at the dose 1 mg/kg (Wager-Srdar et al., 1987).

PF-3845 was dissolved in a vehicle of PEG 400/Tween 80/saline (5%/5%/90%, vol/vol/vol) and was administered IP on alternating days at the dose of 10 mg/kg. All compounds were injected in a volume of 1 mL/kg.

The dose of PF-3845 was chosen on the basis of previous published work demonstrating that systemic administration of PF-3845 increased brain levels of FAAH substrates such as anandamide and oleoylethanolamide (Ahn et al., 2009; Booker et al., 2012; Nasirinezhad et al., 2015; Rock et al., 2015; Henry et al., 2017).

### CAF Diet

The CAF diet consists of mortadella (3.2 kcal/g), cookies (4.8 kcal/g, Macine; Mulino Bianco, Novara, Italy), chocolate muffins (4.5 kcal/g, Mr Day; Vicenzi Group, San Giovanni Lupatoto, Verona, Italy), cheese chips (Fonzies; Mondelez, Milano, Italy; 5.3 kcal/g), cheese (4.3 kcal/g), sippets (5.5 kcal/g; San Carlo, Milano), and lard (9 kcal/g) which were individually weighed before being made available to the rats. Caloric intake from the various foods was calculated based on the nutritional information provided by the manufacturer. Body weight and food intake (expressed as mean kcal/kg ingested ± SEM) were measured daily.

### Experimental Procedure

**Figure 1:**
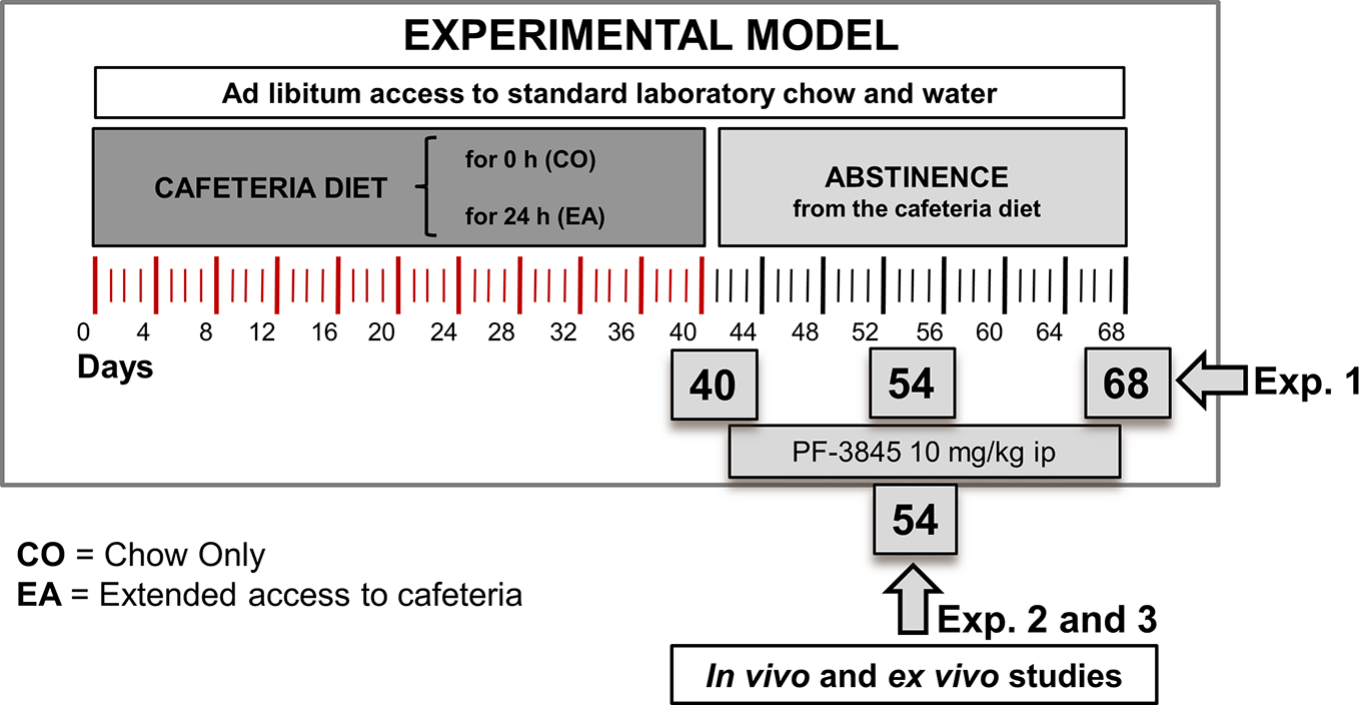
Experimental design for animal model, behavioral studies, and biochemical and molecular analysis. In the first experiment, we evaluated pain threshold following cafeteria diet and during abstinence period. Rats were fed with chow only (CO) or fed 24 h with both chow and extended access to cafeteria diet (EA) for 40 days. On day 41, EA group was fed with only chow until day 68. Pain behavioral tests and Western blot analysis were performed on days 40, 54, and 68. In the second experiment, the CO and EA groups were treated with a selective FAAH inhibitor, PF-3845 (10 mg/kg, IP) from days 41 to 68. Pain behavioral tests were performed on days 54 and 68. Moreover, in the third experiment, at day 54, CB-1 and mu-opioid receptor antagonists were administrated 1 h before performing hot plate test.

In the first experiment (**Figure 1**), we evaluated the pain threshold following CAF diet and during abstinence period. Rats were individually caged and randomly divided into two experimental groups as follows: (1) animals fed with standard chow only (CO) [4RF18; Mucedola, Settimo Milanese, Italy (2.6 kcal/g)] and (2) animals fed with both standard chow and extended access (EA) (24 h/24 h) to CAF diet for 40 days. From day 41, the EA group started a period of abstinence from CAF diet, receiving only chow until day 68, whereas CO rats were maintained with the same chow-only regimen as in the previous 40 days. Pain behavioral tests (hot plate and tail flick) were performed in the morning on days 40, 54, and 68. Rats were sacrificed on days 40 and 68, and their brains collected.

In the following second and third experiments, CO and EA groups were subjected to the same diet protocol described in the first experiment, but during the abstinence they were treated every other day either with vehicle or with the selective FAAH inhibitor, PF-3845 (10 mg/kg, IP) from days 41 to 54.

In the second experiment, the hot plate test was performed on day 54, after which rats were sacrificed, and their brains collected.

Instead, in the third experiment, after PF-3845 treatment, CO and EA rats received on day 54 an acute IP administration of either CB1 or mu-opioid receptor antagonists, SR or Nal, 1 h before the hot plate test was performed.

### Hot Plate Test

Commonly used for evaluating thermal pain sensitivity, the hot plate test provides rapid and precise screening of analgesic drug properties in animals (Le Bars et al., 2001). During the experiment, the rat is introduced into an open-ended cylindrical space with a floor consisting of a plate heated to a constant temperature (55.2°C ± 1°C). As the animal passes through the cylinder, paw licking and jumping, two supraspinally integrated responses, are measured in terms of their reaction times expressed in seconds. This test can only be performed once in each animal, once the jumping response is evaluated. A 60-s cutoff was imposed to avoid tissue damage.

### Tail Flick Test

This nociceptive test used in rodents measures the latency of the avoidance response to thermal stimulus (Le Bars et al., 2001). In the standard method, radiant heat is focused on the dorsal surface of the tail, and the number of seconds it takes until the animal flicks the tail away from the beam is measured. This tail flick latency is a measure of nociceptive sensitivity and spinal nociceptive reflex. A 30-s cutoff was imposed to prevent tissue damage.

### Western Blot Analysis

At days 40, 54, and 68, animals were sacrificed, and whole brains were extracted and homogenized in ice-cold lysis buffer [20 mM Tris–HCl (pH 7.5), 10 mM NaF, 150 mM NaCl, 1% Nonidet P-40, 1 mM phenylmethylsulfonyl fluoride, 1 mM Na_3_VO_4_, leupeptin and trypsin inhibitor 10 μg/mL; 0.25/50 mg tissue]. After 1 h, tissue lysates were obtained by centrifugation at 20,000*g* for 15 min at 4°C. Protein concentrations were estimated by the Bio-Rad protein assay (Bio-Rad Laboratories, Milan, Italy) using bovine serum albumin as standard.

Brain lysate proteins (50 μg) were dissolved in Laemmli sample buffer, boiled for 5 min, and separated by sodium dodecyl sulfate–polyacrylamide gel electrophoresis, and then transferred to a nitrocellulose membrane (240 mA for 40 min at room temperature). The filter was then blocked with 1× phosphate-buffered saline (PBS) and 3% nonfat dried milk for 40 min at room temperature and probed with anti–cannabinoid receptor (CB) 1 (dilution 1:1,000; cat. no. NB120-23703; Novus Biologicals, Cambridge, UK) or anti–mu-opioid receptor antibody (dilution 1:1,000; cat. no. NBP1-96656; Novus Biologicals) in 1× PBS, 3% nonfat dried milk, and 0.1% Tween 20 at 4°C overnight. The secondary antibody was incubated for 1 h at room temperature. Subsequently, the blot was thoroughly washed with PBS and then developed using enhanced chemiluminescence detection reagents (Amersham Pharmacia Biotech, Piscataway, NJ, USA) according to the manufacturer's instructions, and the immune complex was visualized by Image Quant (GE Healthcare, Milan, Italy). The protein bands were scanned and densitometrically analyzed with a model GS-700 imaging densitometer (Bio-Rad Laboratories). To ascertain that blots were loaded with equal amounts of protein lysates, they were also incubated in the presence of the antibody against the β-actin protein (Sigma-Aldrich).

### Statistical Analysis

*In vivo* and *ex vivo* data are presented as mean ± SEM. For the hot plate test and the tail flick test, data were presented as time of response express in seconds. The data were analyzed with analysis of variance (ANOVA) (Systat Software 10.0, San Jose, CA, USA) using the factors described in the Results. We used *post hoc* tests to follow up on significant interaction or main effects (*P* < 0.05) from the factorial ANOVAs. Resistant rats were excluded because they did not significantly increase body weight and did not develop obese phenotype (Cifani et al., 2015).

## Results

### Body Weight

**Figure 2:**
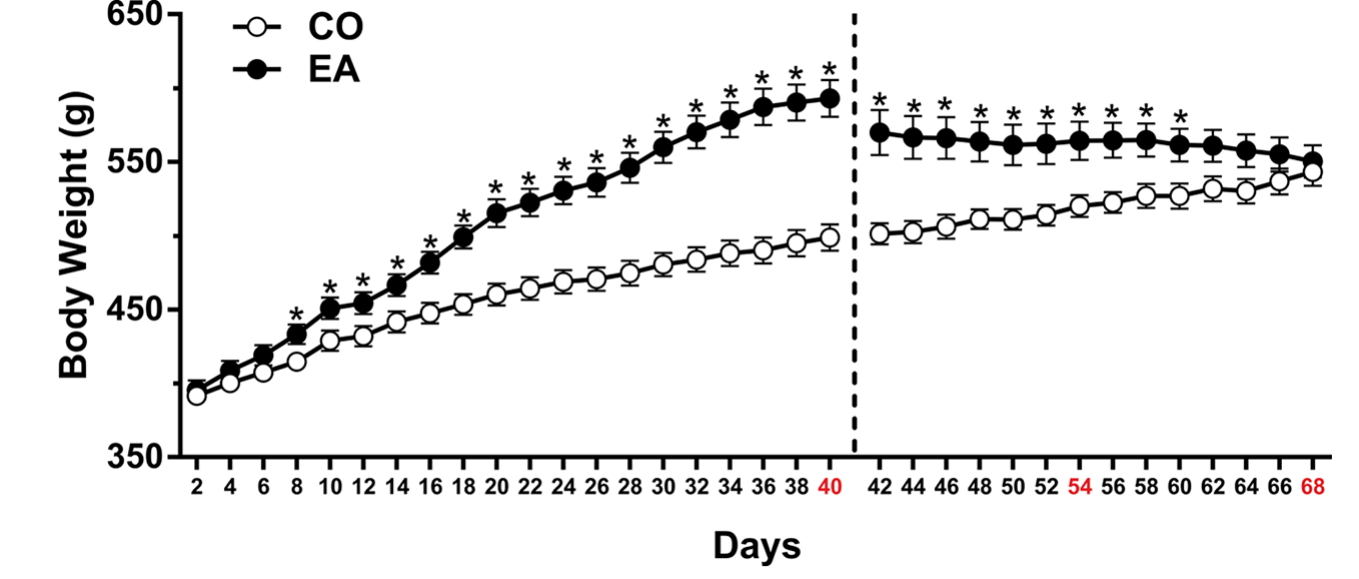
Body weight measured throughout the experimental period. After 8 days, CAF diet significantly increased body weight in EA rats (black spot) if compared to CO rats (white spot) until day 40 (*P < 0.05). During the abstinence period (days 41–68), when only standard chow was available, a progressive and significant decrease in body weight in EA rats was observed. Data are shown as means ± SEM for the CO and EA groups (n = 6).

In the first experiment, the statistical analysis, which included the between-subject factor of diet (CO, EA) and the within-subject factor of time (day), showed a significant difference in body weight (*F*_(1,22)_ = 20.44; *P* < 0.01) and food intake (*F*_(1,22)_ = 985.1; **P < 0.01) between the groups during the 40 days of free access to CAF and/or chow. *Post hoc* tests showed that EA rats immediately increased their food intake (**P < 0.01) (data not shown), and after 8 days of this diet, the body weight of EA rats increased significantly compared to that of CO rats (*P < 0.05) until day 40 (**Figure 2**).

During the abstinence period (days 41–68), when only standard chow was available, EA rats displayed a significant reduction in caloric intake (*F*_(1,10)_ = 144.92; **P < 0.01) and a progressive decrease in body weight (*F*_(1,10)_ = 8.53; *P < 0.05). *Post hoc* tests are shown in **Figure 2**.

In the second and third experiments, EA rats significantly increased both their food intake (data not shown) and body weight in comparison to CO rats during the 40 days of CAF. During the abstinence days, the same reduction in body weight of Experiment 1 was recorded; thus, PF-3845 treatment did not affect feeding behavior (data not shown).

### Pain Evaluation and Involvement of CB1 and Mu-Opioid Receptors After CAF Diet and During Abstinence Period

**Figure 3:**
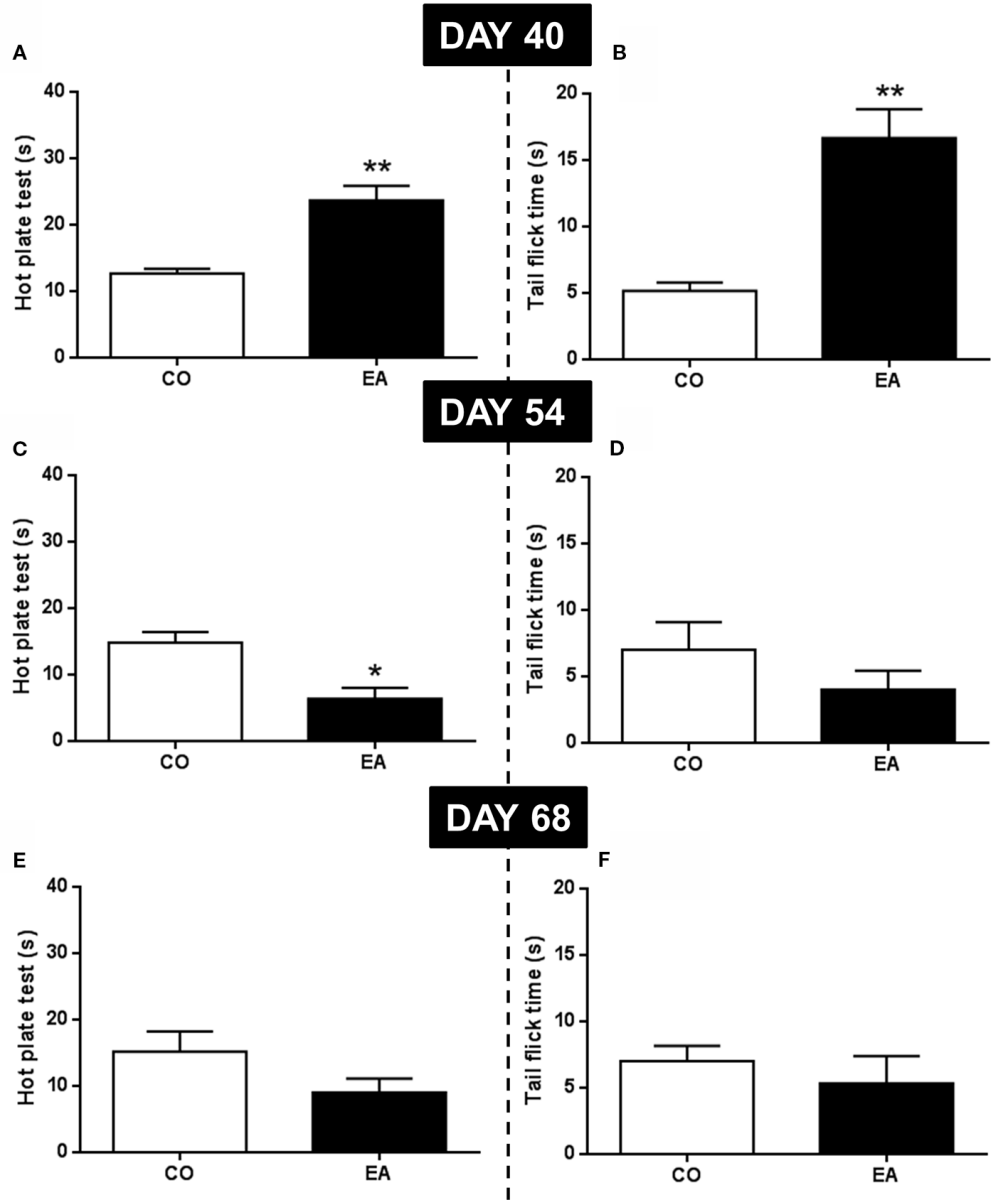
Pain threshold by hot plate and tail flick test were performed at the end of CAF exposure (day 40) and during abstinence period (54 and 68 days). EA rats showed a significant increase of pain threshold both in hot plate **(A)** and tail flick **(B)** tests compared to CO animals (**P < 0.01) at day 40. At day 54, in EA rats, pain sensitivity was significantly increased only in hot plate test (**C**; *P < 0.05). No significant effect was observed in tail flick test **(D)**. At day 68, no difference was observed between EA and CO animals in both tests **(E, F)**. Data are shown as mean ± SEM for the CO and EA groups (n = 6).

In the first behavioral experiment, the pain threshold at the end of the CAF exposure (day 40) and at the middle (day 54) and end (day 68) of the abstinence period was evaluated by hot plate and tail flick tests. At day 40, EA rats showed a significant increase of pain threshold in both the hot plate (*F*_(1,10)_ = 22.59; **P < 0.01) (**Figure 3A**) and the tail flick (*F*_(1,10)_ = 26.07; **P < 0.01) (**Figure 3B**) compared to CO animals. At day 54, in the middle of the abstinence period, EA rats showed increased pain sensitivity, but only in the hot plate test (*F*_(1,10)_ = 13.70; *P < 0.05) (**Figure 3C**), as no significant effect was observed in the tail flick experiment (*F*_(1,10)_ = 1.41; *P* > 0.05) (**Figure 3D**). Finally, at the end of the abstinence period on day 68, no difference was found between EA (*F*_(1,10)_ = 2.78; *P* > 0.05) and CO (*F*_(1,10)_ = 0.50; *P* > 0.05) animals in either test (**Figures 3E, F**).

**Figure 4:**
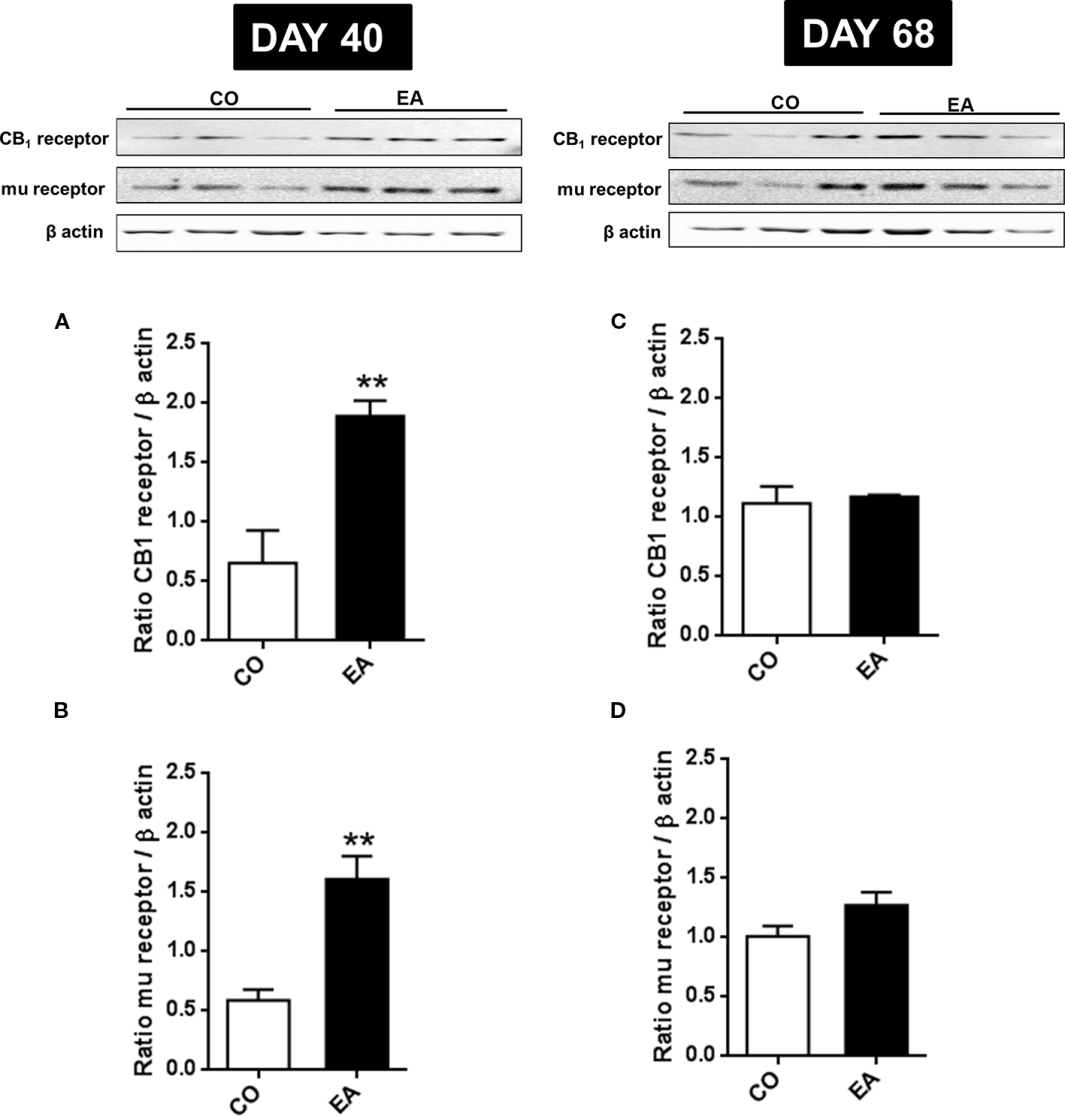
Expression of CB1 **(A, B)** and mu-opioid receptors **(C, D)** in CO and EA rat brain at the end of cafeteria exposure (day 40) and at the end of the abstinence period (day 68). On day 40, a significant increase in CB1 and mu-opioid receptor expression was found in EA rats brains **(A, B)** if compared to CO rats (**P < 0.01). On day 68, no difference was observed between the EA and CO rats **(C, D)**. Representative immunoblots are shown, and densitometric analyses of protein bands of CB1 receptor and mu-opioid receptor are performed on three separate experiments. All data are expressed as mean ± SEM. Equal loading was confirmed by β-actin staining.

In order to correlate the increased pain threshold and the cannabinoid and opioid systems, CB1 and mu-opioid receptors expression was evaluated. At day 40, a significant increase in CB1 (*F*_(1,10)_ = 16.45; **P < 0.01) and mu-opioid (*F*_(1,10)_ = 22.91; **P < 0.01) receptors was found in the brain of EA rats (respectively, **Figures 4A, B**). While the variation in mu-opioid receptor expression has already been reported (Will et al., 2003), this article shows for the first time the correlation between CB1 receptors and highly palatable food.

On day 68, no difference was observed between EA and CO animals in CB1 (*F*_(1,10)_ = 0.15; *P* > 0.05) and mu-opioid receptors (*F*_(1,10)_ = 3.37; *P* > 0.05) (**Figures 4C, D**).

### Effect of PF-3845, A Selective FAAH Inhibitor, on Hypersensitivity by Cafeteria Abstinence and Role of Cannabinergic System

**Figure 5:**
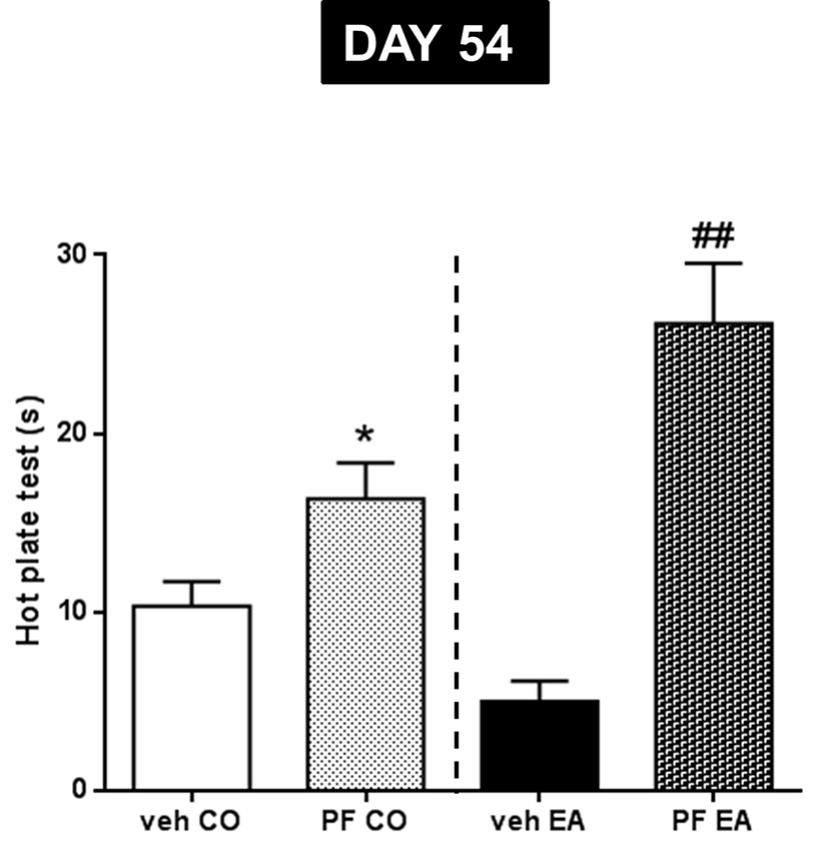
Pain threshold by hot plate test was performed during abstinence period (day 54) after repeated PF-3845 (PF, 10 mg/kg, IP) administration. In CO rats, PF treatment reduced pain sensitivity compared to vehicle CO animals (*P < 0.05). A similar effect was observed in EA rats; in fact, PF treatment increased time of reaction to thermal stimulus compared to vehicle EA animals (^##^*P* < 0.01). Data are shown as mean ± SEM for all groups (n = 6).

The statistical analysis, which included the between-subjects factors of diet (CO, EA) and treatment (vehicle, PF-3845), showed a significant interaction between the two factors (*F*_(1,20)_ = 7.93; *P* < 0.05). Repeated treatment with PF-3845, administered every other day from days 41 to 54 during the second experiment, reduced pain sensitivity in PF-3845–CO rats, as compared to vehicle CO animals, as measured by the hot plate test (**Figure 5**; *P < 0.05). Similar effects were also found in the EA group. In fact, PF-3845–treated animals had a longer time of reaction to thermal stimulus than did vehicle-administered EA animals (**Figure 5**; ^##^ *P* < 0.01)

**Figure 6:**
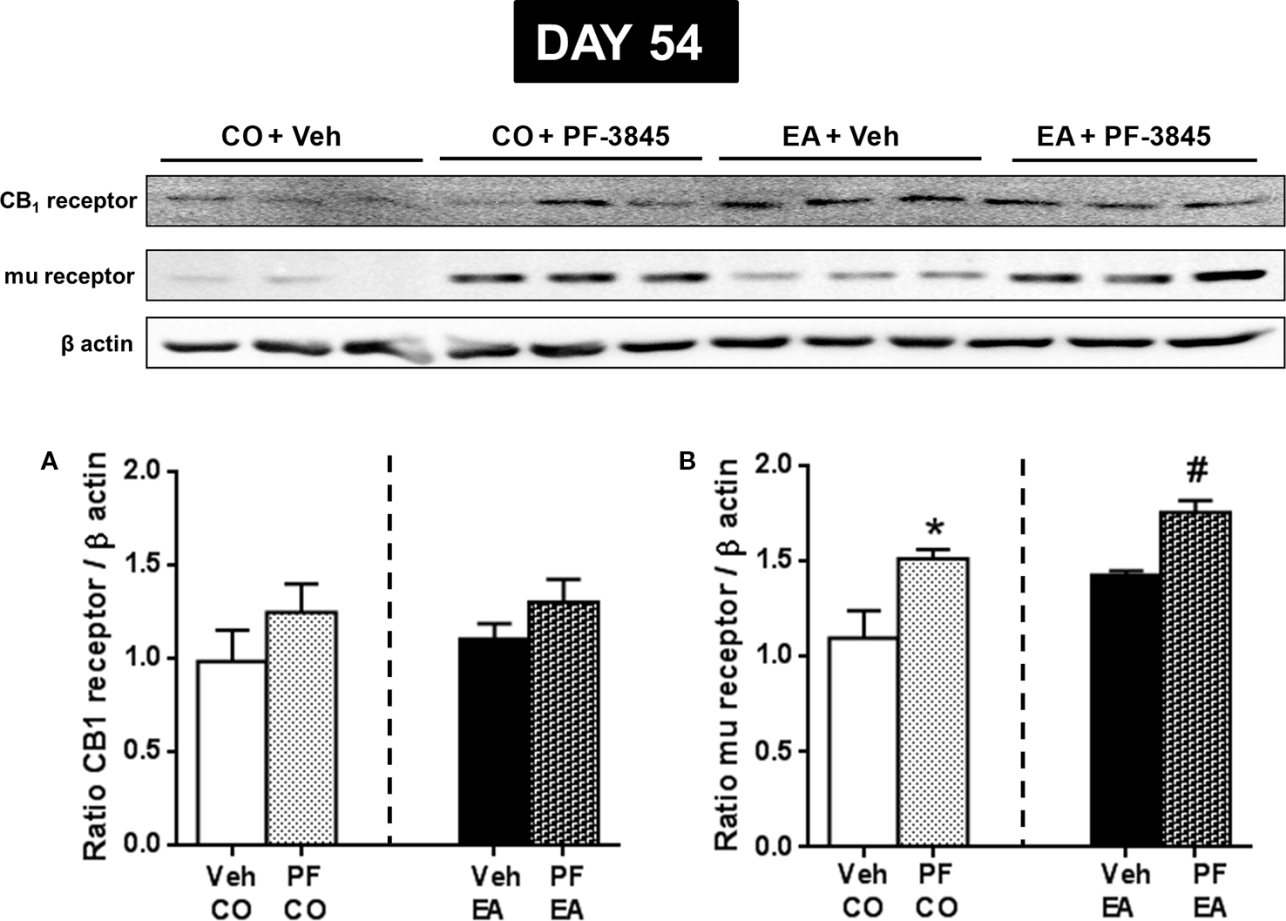
Expression of CB1 **(A)** and mu-opioid receptor **(B)** in CO and EA rat brain after FAAH inhibitor PF treatment at day 54. This drug did not modify CB1 expression **(A)**, whereas mu-opioid receptor expression was significantly increased both in PF-CO and PF-EA rats if compared to their vehicle group (*P < 0.05 vs. CO, ^#^*P* < 0.05 vs. EA) **(B)**. Representative immunoblots are shown, and the densitometric analyses of protein bands of CB1 receptor and mu-opioid receptor are performed on three separate experiments. All data are expressed as mean ± SEM. Equal loading was confirmed by β-actin staining.

Next, we evaluated whether PF-3845 treatment was able to modify CB1 and mu-opioid receptor expression. On day 54, *via* Western blot analysis, we observed that PF-3845 treatment did not modify CB1 expression both in the CO (*F*_(1,10)_ = 1.32; *P* > 0.05) and EA groups (*F*_(1,10)_ = 1.83; *P >* 0.05) (**Figure 6A**), whereas mu-opioid receptor expression was increased in the CO (*F*_(1,10)_ = 7.35; *P* < 0.05) and EA groups (*F*_(1,10)_ = 24.47; *P* < 0.05) (**Figure 6B**).

**Figure 7:**
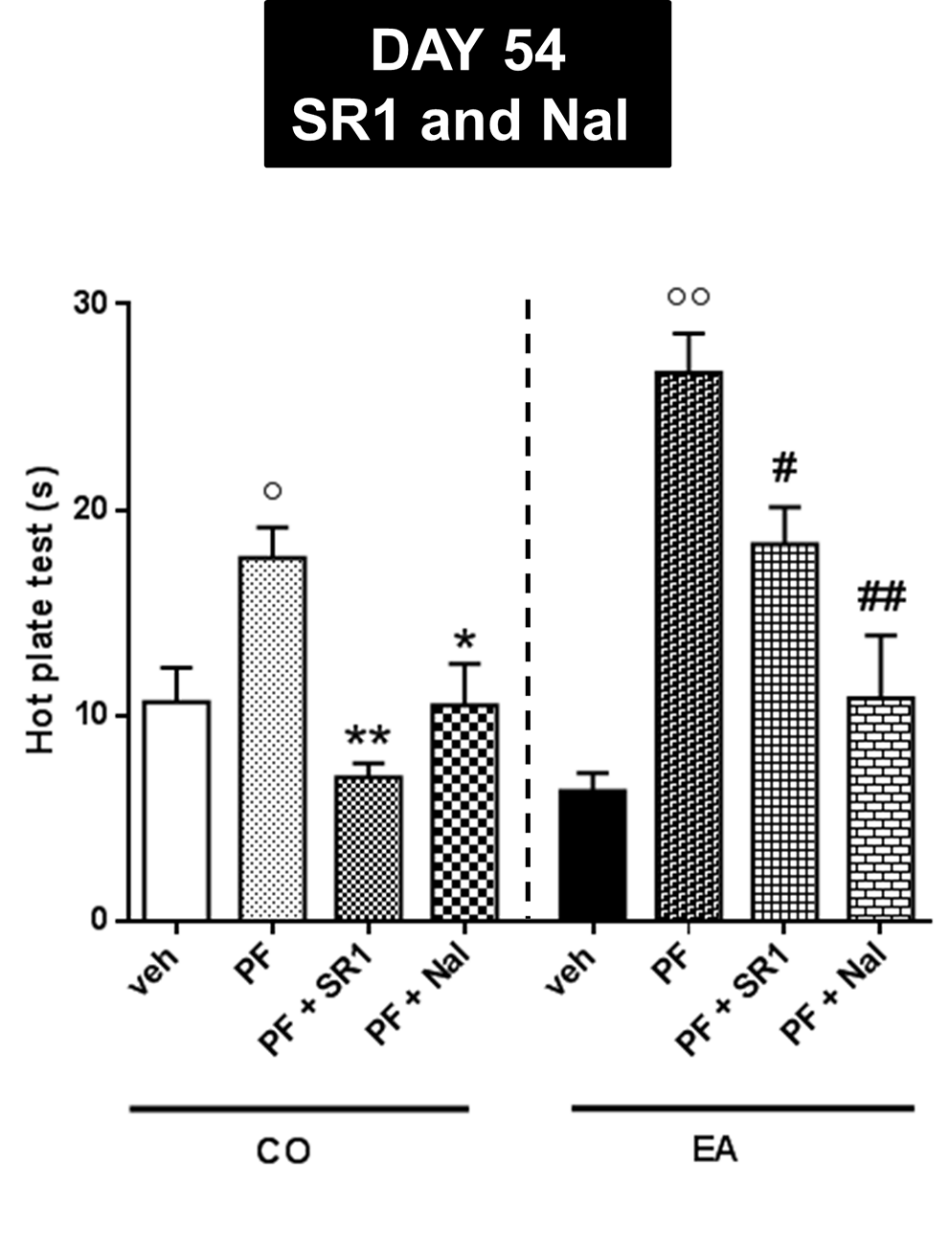
Pain threshold by hot plate test performed at day 54 for evaluating CB1 and opioid receptor involvement. CB1 antagonist, SR1416A (SR1, 1 mg/kg), and an opioid antagonist, naloxone (Nal, 1 mg/kg), were administered in CO and EA rats 1 h before performing hot plate test. In PF-CO and PF-EA rats, treatment with both CB1 and mu-opioid receptors antagonists was able to blunt the effect of FAAH inhibitor (*P < 0.05, **P < 0.01 vs. PF-CO; ^#^ *P* < 0.05 and ^##^ *P* < 0.01 vs. PF-EA; ^°^ *P* < 0.05 and ^°°^ *P* < 0.01 vs. veh). Data are shown as mean ± SEM for all groups (n = 6).

Finally, in order to evaluate CB1 and opioid receptor involvement, at day 54 of the third experiment, we administered a CB1 antagonist/inverse agonist, SR1 (1 mg/kg), or an opioid antagonist, naloxone (Nal, 1 mg/kg) in CO and EA rats. The statistical analysis in the CO group showed a significant effect of the treatment (*F*_(3,20)_ = 8.38; *P* < 0.01). CB1 or mu-opioid antagonists were able to blunt the effect of FAAH inhibitor (**Figure 7**; *P < 0.05 and **P < 0.01). Analysis of variance in the EA group showed a significant effect of the treatment (*F*_(3,20)_ = 18.75; *P* < 0.01). CB1 or mu-opioid antagonist reduced the effect of PF-3845 (**Figure 7**; ^#^ *P* < 0.05 and *^#^* ^#^ *P* < 0.01).

## Discussion

Cannabinoid–opioid receptor cross-talk has been studied extensively, especially regarding the interaction between the molecular and cellular mechanisms in pain threshold (Vigano et al., 2005a; Parolaro et al., 2010), as well as its function in eating behavior (Cota et al., 2006). It is now generally accepted that both systems and the activation of their receptors play a key role in acute and chronic pain modulation (Vigano et al., 2005a; Parolaro et al., 2010; Siuda et al., 2017). Many authors have underlined the important contribution of these two systems in a wide range of physiological processes, including appetite regulation and eating behaviors (Calignano et al., 1998; Pagotto et al., 2006; Cota, 2008). Although it is still unclear whether obesity can alter pain sensitivity (Hitt et al., 2007; Somers et al., 2011), several authors have shown a correlation between an increase of body weight and a significant alteration of pain sensitivity (Torensma et al., 2016; Chin et al., 2019). Obesity is usually associated with musculoskeletal system disorders such as back pain or osteoarthritis. Moreover, many studies indicate that it could be one of the risk factors in some nociceptive pain conditions, and more recently, it has been demonstrated that obesity is associated with worsening in neuropathic pain intensity (Hozumi et al., 2016). On the other hand, patients with intense chronic pain more frequently suffer from eating disorders (Godfrey et al., 2018) and eat more sweet-tasting food when they feel pain, particularly if they are not able to control their impulses (Darbor et al., 2017). In recent years, more attention has been devoted to obesity and to the behavioral alterations due to continuous and compulsive consumption of highly palatable foods (Zheng and Berthoud, 2007). In fact, obesity from palatable diet induces a change in different neurotransmitter systems leading to changes in several CNS areas, such as the Ventral Tegmental Area (VTA), hypothalamus, and Nucleus Accumbens (NAc), the same zones involved in the reward system (Sampey et al., 2011). In this context, the CAF diet is a valid tool for imitating the contemporary diet, which is characterized by high salt content and high-calorie fatty and sugary foods (Johnson and Kenny, 2010; Sampey et al., 2011). Extended exposure to this kind of diet induces a significant modification of several signaling pathways related to physiological control, such as food intake and stimulation of the reward system. This suggests that palatable foods could lead to addiction through mechanisms that are similar to those in drug abuse (Pelchat, 2002; Wang et al., 2004; Johnson and Kenny, 2010). In this scenario, opioid and cannabinergic systems are significantly involved (Zhang et al., 2003; Erlanson-Albertsson, 2005).

The aim of our study was not only to study the correlation between obesity and pain in rats with an extended access to palatable foods, a topic still debated (Hitt et al., 2007; Somers et al., 2011), but also to evaluate the responses to pain during the period of abstinence from a highly palatable diet (Sampey et al., 2011). In particular, we studied (i) the difference in pain perception between animals fed for 40 days with extended access to CAF (EA rats) and rats fed with standard chow (CO rats); (ii) the contribution of the opioidergic and cannabinergic systems, through the involvement of CB1 and mu-opioid receptors in the brain. Results clearly indicate that, at the end of the CAF diet, EA animals showed a significant increase in pain threshold compared to rats fed with standard diet, perhaps due to an up-regulation of CB1 and mu-opioid receptors. Surprisingly, in this study, we have shown that palatable food increased CB1 receptor expression in the brain, suggesting that the observed analgesic effect was probably mediated by both the opioid and the cannabinoid systems.

Our results are in agreement with previous reports in which obese subjects were less sensitive to painful stimuli (Zahorska-Markiewicz et al., 1983; Zahorska-Markiewicz et al., 1988; Khimich, 1997). Taking into account that endogenous opioid levels were increased in obese humans (Givens et al., 1980; Cozzolino et al., 1996; Karayiannakis et al., 1998; Martinez-Guisasola et al., 2001) and animals (Smith et al., 2002; Barnes et al., 2003), the decrease in pain sensitivity observed here may be associated with these changes.

Regarding the CB1 receptor, studies have shown that CB1 antagonists reduce the intake of palatable sugar in rats (Gardner, 2005; Mathes et al., 2008) and prevent the orexigenic effect of the endocannabinoid agonist anandamide on food intake (Cota et al., 2006). Furthermore, endocannabinoids induce a neuromodulation in specific brain areas (e.g., NAc), and this neurochemical response contributes to subjective reward and positive reinforcement. These findings support a role of endocannabinoids in the hedonistic effects of natural rewards such as palatable food in eating disorders (Gardner, 2005; Parsons and Hurd, 2015; Pucci et al., 2016; Pucci et al., 2018; Pucci et al., 2019). We have also evaluated pain sensitivity after withdrawal of CAF diet, because deprivation of highly palatable food induces a neuroadaptive response associated with behavioral alteration (depression and anxiety) (Johnson and Kenny, 2010; de Macedo et al., 2016). Fourteen days after the end of the CAF diet (day 54), we observed a weak increase of pain sensitivity, which was significant in the hot plate test, but not in the tail flick test. These different results may suggest that there were alterations in the brain rather than in the spinal cord. In fact, it is well known that the hot plate test is a classic pain model for supraspinal stimuli, whereas the tail flick test is a model for spinal stimuli (Langerman et al., 1995). In agreement with these observations, Western blot analysis showed a slight increase of mu-opioid receptor expression in the brain of rats that previously had extended access to the CAF diet, but no difference was observed in spinal cord tissues between these two groups (data not shown).

These findings help to explain the results of our study of standard chow intake during abstinence from the CAF diet; ingestion behavior alone is probably insufficient to elicit analgesia, and instead the hedonic value from palatable food may be needed to inhibit the response to pain (Foo and Mason, 2009). Indeed, de Freitas et al. (2012) found that acute oral administration of sucrose can induce antinociception mediated by the actions of endogenous opioid peptide and mu-opioid receptor (de Freitas et al., 2012).

Opioids and cannabinoids share several pharmacological actions that may be relevant in understanding the therapeutic potential of cannabinoids, particularly as analgesics (Manzanares et al., 1999). These two systems are widely distributed throughout the CNS and have key role in both reward-related feeding (Cota et al., 2006; Pagotto et al., 2006) and modulating pain thresholds. The strong relationship between cannabinergic and opioidergic systems and the capability of a system to activate the other one is well known; in fact, CB1 agonists induce antinociception increasing opioid precursors' gene expression or releasing endogenous opioids (Houser et al., 2000; Valverde et al., 2001). It has be shown that extended treatment with a cannabinoid agonist induced a significant increase of mRNA levels of endogenous precursor opioid peptides in several CNS areas (Corchero et al., 1997a; Manzanares et al., 1998) and that CB1 agonist can induce down regulation of its receptor and enhance brain reward function producing reward effects (Daigle et al., 2008). On the other hand, it has been suggested that opioid receptor–mediated analgesia might be enhanced by an increase in endocannabinoid levels (Bushlin et al., 2010), because morphine-induced analgesia was enhanced by FAAH initiator in the CNS, as already reported (Pacheco Dda et al., 2009).

For these reasons, in this study, we used an indirect agonist, the selective endocannabinoid clearance inhibitor, PF-3845. Specifically, this FAAH inhibitor was used to evaluate the contribution of the CB1 receptor to pain perception during an abstinence period from the CAF diet. Our results clearly showed that repeated PF-3845 treatment decreased pain sensitivity in CO and EA rats compared to the respective vehicle groups. Surprisingly, a significant effect between in abstinence (EA + PF-3845) and normal (CO + PF-3845) rats was observed. This activity was mainly mu-opioid receptor mediated: Western blot analysis showed a significant up-regulation of this receptor following PF-3845 treatment. In support of our data, Vigano et al. (2005b) showed that repeated treatment with CB1 agonist significantly increased mu-opioid receptor density in the lateral hypothalamus and in the periaqueductal gray. Finally, we performed behavioral experiments to study pain, using both CB1 and opioid antagonists (SR1 and naloxone, respectively) to confirm our hypothesis. In fact, results showed that pretreatment with cannabinoid and even better opioid antagonists reduced PF-3845–increased sensitivity.

Our findings point out that an extended exposure to palatable food followed by abstinence from it induced a significant change in pain perception, leading to increased pain sensitivity. In this scenario, the cannabinergic system played a key role in regulating, “compulsive” food intake behavior and pain response.

Further work is necessary in this field to improve our understanding of the modulation of pain sensitivity in obese subjects. This is extremely important in order to plan appropriate pain management strategies for overweight patients, further considering that obesity is a rapidly growing a global pandemic problem (Collaborators et al., 2017; Chooi et al., 2019).

## Data Availability Statement

The datasets generated for this study are available on request to the corresponding authors.

## Ethics Statement

The animal study was reviewed and approved by the Ministry of Health, protocol n.887/2015-PR; 19/01/2017.

## Author Contributions

Participated in research design: AC, SG, CCi, MM, and RR. Conducted experiments: MM, CCi, CA, MG, CD, CCr, GL, EM, AR, and LB. Performed data analysis: MM, CCi, CA, and RR. Wrote or contributed to the writing of the manuscript: MM, CA, CCi, and RR.

## Funding

This work was supported by Italian Ministry of University and Research grant (PRIN2012 prot. #2012JTX3KL) to CCi, RR, and SG.

## Conflict of Interest

The authors declare that the research was conducted in the absence of any commercial or financial relationships that could be construed as a potential conflict of interest.
